# Awareness of human papillomavirus and factors associated with intention to obtain HPV vaccination among Korean youth: quasi experimental study

**DOI:** 10.1186/s12914-015-0042-2

**Published:** 2015-02-21

**Authors:** Hae Won Kim

**Affiliations:** The Research Institute of Nursing Science, College of Nursing, Seoul National University, Taehakro 103, Jongro-Gu Seoul, South Korea 110-799

**Keywords:** HPV, Vaccination, Education, Youth

## Abstract

**Background:**

This study aimed to determine the awareness among fifth-grade girls and boys of sexually transmitted diseases (STDs), cancer, and human papillomavirus (HPV), and to determine the factors associated with intention to obtain the HPV vaccination.

**Methods:**

A quasi experimental design was employed with Korean fifth-grade students as the subjects for this study (*n*=117). Prior to providing HPV education, the awareness and health beliefs regarding STDs and cancer prevention were assessed according to gender. After 2 hours of HPV education, gender comparisons were made with respect to the awareness and health beliefs, HPV knowledge, and intention to obtain the HPV vaccination, and the factors associated with that intention.

**Results:**

Prior to the 2hours education session, only two boys knew that HPV is a virus. There were significant gender differences with respect to responses to the statements “STD is preventable” (*χ*^2^=8.76, *p*=0.013) and “cancer is preventable” (*χ*^2^=6.37, *p*=0.041), and concerns about the pain associated with vaccine injection (*z*=−2.44, *p*=0.015). After HPV education, there were no significant gender differences in HPV knowledge and intention to obtain the HPV vaccination. Awareness that “HPV vaccine can prevent cervical cancer” was significantly related to intention to obtain the HPV vaccine among both boys and girls.

**Conclusions:**

Increased HPV knowledge could positively influence the intention to obtain the HPV vaccination among youth. Thus, HPV education at elementary school would be helpful to make students aware of HPV and the importance of HPV prevention.

**Electronic supplementary material:**

The online version of this article (doi:10.1186/s12914-015-0042-2) contains supplementary material, which is available to authorized users.

## Background

The recognition that human papillomavirus (HPV) infection is associated with a high risk of cervical cancer has resulted in a paradigmatic shift in the focus of cervical cancer prevention from female adults to youth, with the introduction of a prophylactic vaccination against HPV [1]. The key to preventing human papillomavirus (HPV) infection is education and immunization of youth [2]. In childhood, HPV infection appears to be the principal cause of common warts, genital warts, recurrent respiratory papillomatosis, and both low- and high-grade squamous intraepithelial lesions [3]. In a prospective cohort study among Dutch children in grades 1–7 (aged 4–12 years), the incidence of warts was 29 per 100 person-years. Thus, preventive efforts should focus on limiting HPV transmission in the school setting [4] (Additional file 1).

There are few statistical data pertaining to sexually transmitted diseases (STDs) in Korea, and monitoring of the prevalence of STDs in the past has relied on reports from a sentinel surveillance system. The findings from such reports have led to genital warts (condyloma accuminata), which are caused by HPV infection, being officially designated as one of the seven communicable infections since 2001 [5]. The Korean Society of Gynecology Oncology reported that the prevalence of these warts increased from 2.1% in 2004 to 16.5% in 2011, and was highest for those younger than 30 years [6]. One study found that the incidence of STDs in Korea among those aged 15–29 years accounted for 40% of the incidence in the entire population of Korea, and the incidence of genital warts in particular was estimated to be almost five times higher in 2011 than in 2001 [7]. In 2010, it was estimated that on average 5.3% of Korean adolescents were sexually active. Of these, 10% had experienced an STD, and 44.7% of those with an STD had not received treatment for it. It is also noteworthy that the proportion of sexually active middle-school students increased from 0.5~1.5% in 2007 to 2.3~3.1% in 2010 [7,8]. These facts have led to a change in the focus of strategies designed to prevent STDs, including HPV-related infections (mainly warts), so that they are directed toward young Korean adolescents rather than adults.

With respect to Western countries, it has been found that awareness of HPV and the HPV vaccination was relatively low among Hungarian adolescents, and that most of the included students were receptive to receiving further information about sexually transmitted diseases (STDs) [9]. There also appears to be a lack of awareness of HPV associated with uncertainty regarding the decision-making process to obtain the HPV vaccination among male adolescents in the UK, [10] and a general concern regarding the safety of the HPV vaccine, largely due to their unfamiliarity with it [11]. Despite the increasing assertions that men should be actively involved in HPV prevention [12-14], there remains limited information promoting sexual health among boys [15]. For example, in Australia, even though an HPV vaccination program for girls has been implemented in schools, one study found that the level of understanding about HPV remained low among both adolescents and their parents [16]. Furthermore, the uptake of the HPV vaccination among boys was low, and was dependent upon the school’s geographical location and socioeconomic status [17].

On the other hand, a study conducted in Korea found that adolescents demonstrated an apparent lack of awareness of the HPV vaccine and related health beliefs, and their HPV vaccination rates were reportedly as low as 1.3% among boys and 9.5% among girls [18]. Although recommended age ranges for HPV vaccination in Korea have been established as 9–26 years for girls and 9–15 years for boys, a school-based HPV vaccine program has yet to be introduced in this country, and so the cost of the vaccination must be borne entirely by their parents [12,19].

Since 2009, Korean legislation has stipulated that school health education disseminated by health teachers must be provided to every fifth- and sixth-grade student [20]. Until now, sex education focusing on HPV prevention has not been available in Korea, in part because of the school policies and the attitudes or beliefs of the school teacher [12]. The total amount of time allocated to sex education in Korea is 10 hours per year throughout the whole curriculum, and since only 10~15% of schools throughout the nation employ qualified health teachers, this education is not delivered in dedicated health education classes (school nurses in Korea are able to teach about STDs) [7].

However, it has been shown that while most (89.3%) Korean health teachers were aware of the necessity to provide HPV education to their students, there was no formal HPV education program for them to follow [12] and little is known about HPV awareness and attitudes toward the HPV vaccination among Korean youth.

Based on the aforementioned findings, it is necessary to explore the basic understanding of HPV among the Korean youth, and to develop a preliminary HPV education program for Korean health teachers to apply to fifth- and sixth-grade students. The present study applied the Health Belief Model (HBM) to assess the attitudes and intentions among Korean adolescents related to STDs and cancer prevention, which are thought to be associated with intention to obtain the HPV vaccination [12,21], and used that HBM framework to determine what is required to increase their intention to obtain an HPV vaccination. In addition, previous studies have found gender differences with respect to awareness of HPV, and attitudes and the intention toward HPV vaccination among Korean adolescents, university students, and adults [14,18,22]. Thus, HPV education should be gender based and should commence from the primary school setting, so that both genders can be taught that HPV is a matter of concern for both women and men, raising awareness of their equal responsibility for HPV prevention.

The aim of this study was to determine the awareness and health beliefs of Korean fifth-graders regarding STDs, cancer, and HPV infection both before and after delivery of a 2-h HPV education program. In the first instance, the awareness and health beliefs regarding STDs and cancer among Korean fifth-grade girls and boys were assessed by survey. After delivery of the HPV education program to all of the subjects, gender differences in awareness and health beliefs, HPV knowledge, and intention to obtain the HPV vaccination were examined, as well as the factors associated with that intention.

## Methods

### Research design

A quasi experimental study was employed to determine the HPV awareness and HPV vaccination intention among fifth-grade girls and boys, and the effect on those parameters of a 2-h HPV education program.

### Participants and setting

Convenience sampling was employed to recruit the study participants. This study included all fifth-grade students (*n*=117), both girls (*n*=47) and boys (*n*=70), from an elementary school located in Gangneung city, Korea. Prior to recruitment, permission to access the students was obtained from the school principal, health teacher, and two teachers engaged in fifth-grade teaching. Gangneung city is in the Kang Won Do province, which is one of the eight administrative regions in Korea, and is located on the eastern coast of the country; it is approximately 2.5 hours drive from Korea’s capital, Seoul. In 2013 there were 37 primary schools in Gangneung city, with a total of 11,283 students. The setting for this study was selected by recommendation, mainly because all of the fifth-grade students there were able to participate. The selected school comprised 21 classes, with a total of 506 students overall and 117 fifth-grade students.

### HPV education

Prior to HPV education, the relevance of the teaching content and the methodologies were evaluated by two HPV professionals and a health teacher from the elementary school. The following specific topics were included: (1) HPV as a virus that causes cervical, penile, and oral cancers, and STDs such as warts; (2) low- and high-risk types of HPV infection; (3) the purposes and benefits of, and barriers toward obtaining the HPV vaccination; (4) HPV vaccination of girls and boys for preventing genital warts and genital cancers; and (5) early prevention of STDs and cancers caused by HPV for both genders (e.g., condom use, delayed sexual debut, genital observation, and vaccination). The underlying concept of HPV education in this study was that it should be gender based, so that it was fundamentally designed with a view to promoting gender equality regarding HPV prevention. It was intended that both the girls and boys would become aware of their mutually dependent responsibility regarding HPV prevention [22].

The HPV education program comprised two 50-minute sessions that were applied during the period 9.00 a.m. to 1.00 p.m. in the classroom consecutively on the same day. The program comprised a lecture, a PowerPoint presentation, and a discussion. Two coeducational classes comprising 57 and 60 students participated in the HPV education program. The two classes were not allowed to communicate with each other during the program, so that while one class was taking the HPV education session in the classroom, the other class was performing extracurricular activities outside the classroom. A handout for the lecture was given to all students to take home.

### Data collection procedure

The data were collected in October 2011 in the classroom before and after HPV education using self-administered questionnaires. The study was conducted in the two classrooms by the research team. There were no dropouts between the pre- and posttests. The research protocol was approved by the Myoungji University Hospital Institutional Review Board. Permissions from the parents and the students were not sought because the school principal and teachers all concluded that it was not necessary for this study. At the first meeting in the classroom, the health teacher and the researchers explained the purposes, content, and schedule for the study, and informed the subjects that the data they provided would be confidential and anonymous. The pre- and posttest were conducted prior to and immediately after delivery of the HPV education, respectively. After the posttest, the students received a small gift, costing approximately US$ 3 (3,500 Korean won), for successfully completing the study.

### Measures

The contents and constructs of the structured questionnaire were validated by three experts in HPV research and sex education, and were tested on ten fifth-grade girls and boys aged 12 years. In the pretest, general information was gathered, including age, sex, feelings regarding the vaccination, awareness of the necessity for the vaccination, whether they had heard of HPV or any other viruses, and general awareness of STDs and cancer. The HBM variables were also assessed. In the posttest, HPV knowledge and intention to obtain the HPV vaccination were added to the survey.

The HBM used in this study measures attitudes toward STDs and cancer prevention, and intention to obtain a vaccination to prevent STDs and cancer, and was modified to fit the present study sample based on the findings of previous studies [12,14]. The four main variables of the theoretical constructs of the HBM were selected in the present study since they were the first to be developed and research has confirmed their usefulness in preventing HPV infection [3,12,23].

Items pertaining to HBM variables consisted of perceived severity (two items), perceived barriers (one item), perceived benefits (two items), and intention to obtain the vaccination (two items); the content of all four variables was validated by HBM researchers. The students recorded their level of agreement on a 5-point scale ranging from 1 (“not at all”) to 5 (“very much”). A Cronbach’s α of 0.78 for the HBM scales used in the present study confirmed their reliability.

In the posttest, general awareness of STDs and cancer, and HBM variables were evaluated. Moreover, intention to obtain the HPV vaccination was assessed on a 5-point scale ranging from 1 (“not at all”) to 5 (“very much”), and HPV knowledge was assessed by eight items that also had been modified to fit the students in this study based on the findings of previous studies [24,25]. A Cronbach’s α of 0.85 for the HPV knowledge test used in this study confirmed its reliability. A score of 1 was assigned to each item for a correct answer, and score of 0 was assigned for an incorrect or “do not know” answer.

### Data analysis

All variables were analyzed using frequencies, means, proportions, standard deviations, and percentages. At the pretest, the homogeneity test of the general awareness of STDs and cancer, and HBM variables between girls and boys was analyzed using the *t* and *χ*^2^ tests. At the posttest, gender differences in measurement variables were analyzed using the *χ*^2^ test, the Mann–Whitney *U* test, and the Wilcoxon signed-rank test. Factors associated with intention to obtain the HPV vaccination were analyzed using the Spearman rho coefficient. IBM SPSS v. 20.0 statistics software was used for all statistical analyses.

## Results

Of the 117 fifth-grade students enrolled in this study, 59.8% were boys (*n*=70) and 40.2% were girls (*n*=47), and all were aged 11–12 years. Before the HPV education session (i.e., pretest), there were significant differences regarding feelings about the vaccine injection (*χ*^2^=7.66, *p*=0.006) and awareness of the virus (*χ*^2^=6.12, *p*=0.013). When asked whether they knew about any viruses, 17.5% of the boys and 34.0% of the girls answered that they did not know, or gave incorrect answers, including referring to bacteria and computer viruses. The most commonly known viruses were flu, influenza, and H1N1 among both boys and girls (Table 1).

**Table 1:** **Gender comparisons of the general characteristics at pretest**

**Variable**	**Categories**	**Boys (** ***n***=**70)**	**Girls (** ***n***=**47)**	***χ*** ^**2**^ **or** ***t*** **(** ***p*** **)**
		***n*** **(%) or mean**=**SD**		
Age (years)		11.97±0.17	12.00±0.00	1.36 (0.246)
	11	1 (2.9)	0 (0.0)	
	12	68 (97.1)	47 (100.0)	
Feelings about vaccine injection (*n*=115)	Afraid	6 (8.7)	13 (28.3)	7.66 (0.006)
	Not afraid	63 (91.3)	33 (71.1)	
Need for vaccination	Know	60 (85.7)	39 (83.0)	0.16 (0.688)
	Don’t know	10 (14.3)	8 (17.0)	
Heard of viruses (*n*=114)	Yes	7 (10.3)	13 (28.3)	6.12 (0.013)
	No	61 (89.7)	33 (71.7)	
Known viruses^a^	Flu/influenza	15 (14.6)	10 (20.0)	
	H1N1	51 (49.5)	16 (32.0)	
	Avian flu	1 (1.0)	-	
	Encephalitis	4 (3.9)	1 (2.0)	
	Hepatitis	5 (4.9)	-	
	Tetanus	2 (1.9)	-	
	Smallpox	2 (1.9)	-	
	Varicella	1 (1.0)	2 (4.0)	
	Ebola	1 (1.0)	-	
	HPV	2 (1.9)	-	
	STD	-	1 (2.0)	
	Pneumonia	-	2 (4.0)	
	Rabies	-	1 (2.0)	
	Don’t know or misunderstand	18 (17.5)	17 (34.0)	

^a^Multiple responses possible.

STD, sexually transmitted disease; HPV, human papillomavirus.

The data presented in Table 2 reveal significant gender differences with regard to the items “STD is preventable” (*χ*^2^=8.76, *p*=0.013) and “cancer is preventable” (*χ*^2^=6.37, *p*=0.041) at pretest, but there were no significant gender differences regarding awareness of STDs and cancer prevention at posttest.

**Table 2:** **Gender comparisons of awareness of STDs and cancer prevention between pre- and posttest**

**Awareness of STD and cancer prevention**		**Boys (pre)**	**Girls (pre)**		**Boys (post)**	**Girls (post)**	
		***n*** **(%)**		***χ*** ^**2**^ **(** ***p*** **)**	***n*** **(%)**		***χ*** ^**2**^ **(** ***p*** **)**
Viral infection is preventable	Yes	49 (70.0)	30 (63.8)	1.14 (0.566)	63 (90.0)	45 (97.8)	2.65 (0.104)
	No	1 (1.4)	2 (4.3)		0 (0)	0 (0)	
	Don’t know	20 (28.6)	15 (31.9)		7 (10.0)	1 (2.2)	
STDs are preventable	Yes	25 (35.7)	6 (12.8)	8.76 (0.013)	64 (91.4)	42 (93.3)	2.24 (0.327)
	No	0 (0)	1 (2.1)		0 (0)	1 (2.2)	
	Don’t know	45964.3)	40 (85.1)		6 (8.6)	2 (4.4)	
Cancer is preventable	Yes	43 (61.4)	18 (38.3)	6.37 (0.041)	60 (85.7)	41 (89.1)	2.03 (0.369)
	No	4 (5.7)	6 (12.8)		1 (1.4)	2 (4.3)	
	Don’t know	23 (32.9)	23 (48.9)		9 (12.9)	3 (6.5)	
Time to begin STD prevention	Onset of puberty	33 (61.1)	29 (72.5)	1.67 (0.434)	67 (95.7)	45 (97.8)	0.37 (0.542)
	Adulthood	11 (20.4)	7 (17.5)		3 (4.3)	1 (2.2)	
	Not necessary	10 (18.5)	4 (10.0)		0 (0)	0 (0)	
Time to begin cancer prevention	Onset of puberty	28 (45.2)	23 (50.0)	0.78 (0.677)	49 (70.0)	36 (78.3)	0.97 (0.325)
	Adulthood	27 (43.5)	20 (43.5)		21 (30.0)	10 (21.7)	
	Not necessary	7 (11.3)	3 (6.5)		0 (0.0)	0 (0.0)	
Methods of STD prevention^a^	Abstinence	0 (0.0)	1 (2.1)	-	9 (7.9)	8 (10.7)	-
	Condom use	2 (2.9)	0 (0.0)		39 (34.2)	22 (29.3)	
	Genital examination	1 (1.4)	1 (2.1)		22 (19.3)	16 (21.3)	
	Hygiene habits	7 (10.0)	6 (12.8)		30 (26.3)	16 (21.3)	
	None	3 (4.3)	1 (2.1)		3 (2.6)	1 (1.3)	
	Don’t know	53 (75.7)	37 (78.7)		11 (9.6)	12 (16.0)	

^a^Multiple response.

Pre, pretest; post, posttest.

STD, sexually transmitted disease.

Significant gender differences were obtained for “concern about pain at vaccine injection” at both pretest (*χ*^2^=−2.44, *p*=0.015) and posttest (*χ*^2^=−2.31, *p*=0.021; Table 3). There were significant effects for boys at posttest with regard to the HBM variables, except for intention to obtain the HPV vaccination for cancer prevention. However, there were significant effects on HBM variables among the girls, with the exception of severity of cancer and “concern about pain at vaccine injection.” At the completion of the HPV education (i.e., posttest), there were no significant gender differences with regard to intention to obtain the HPV vaccination and HPV knowledge scores (Table 4). Regarding factors associated with intention to obtain the HPV vaccination, “concern about pain at vaccine injection” was significant only for the girls. However, the knowledge that “HPV vaccine prevents cervical cancer” was significantly associated with the intention to obtain the HPV vaccination among both boys (*r*=0.25, *p*=0.037) and girls (*r*=0.13, *p*=0.037; Table 5).

**Table 3:** **Gender comparisons of health-belief variables related to STDs and cancer prevention between pre- and posttest**

**Health-belief variables related to STDs and cancer prevention (range, 1–5)**	**Boys (pre)**	**Girls (pre)**	**Z** ^*^ **(** ***p*** **)**	**Boys (post)**	**Girls (post)**	**Z** ^*^ **(** ***p*** **)**	**Boys**	**Girls**
							**(pre–post)**	**(pre–post)**
	**Mean** **±** **SD**			**Mean** **±** **SD**			**Z** ^**ϯ**^ **(** ***p*** **)**	**Z** ^**ϯ**^ **(** ***p*** **)**
Severity of STD	2.94±0.85	3.07±0.65	−1.14 (0.255)	3.81±0.97	3.64±0.71	−1.08 (0.281)	−5.53 (<0.001)	−3.67 (<0.001)
Severity of cancer	3.99±1.20	4.04±0.95	−0.25 (0.806)	4.30±0.86	4.28±0.58	−0.79 (0.432)	−2.38 (0.017)	−1.82 (0.069)
Intention to obtain injection for STD prevention	3.52±0.85	3.28±0.93	−1.14 (0.253)	4.11±0.81	4.26±0.57	−0.76 (0.445)	−5.08 (<0.001)	−4.85 (<0.001)
Intention to obtain injection for cancer prevention	4.23±0.79	4.09±0.80	−1.08 (0.282)	4.30±0.69	4.37±0.57	−0.35 (0.723)	−1.24 (0.216)	−2.81 (0.005)
Concern about pain at vaccine injection^a^	3.09±1.04	2.62±1.05	−2.44 (0.015)	3.29±1.13	2.76±1.10	−2.31 (0.021)	−2.21 (0.026)	−1.11 (0.268)
Benefit of STD prevention injection	3.59±0.87	3.54±0.75	−0.10 (0.921)	4.19±0.73	4.07±0.58	−1.09 (0.278)	−4.68 (<0.001)	−3.76 (<0.001)
Benefit of cancer prevention injection	4.03±0.93	3.98±0.74	−0.58 (0.564)	4.32±0.74	4.31±0.51	−0.57 (0.570)	−3.52 (<0.001)	−2.80 (0.005)
Cronbach’s α for seven items=0.78								

^a^Reverse coding.

^*^Mann–Whitney *U* test, ^ϯ^Wilcoxon signed-rank test.

STD, sexually transmitted disease.

**Table 4:** **Gender comparisons of intention to obtain HPV vaccination and HPV knowledge at posttest**

	**Boys**	**Girls**	***χ*** ^**2**^ **or** ***Z*** ^*^ **(** ***p*** **)**
	***n*** **(%) or mean**±**SD**		
Intention to obtain HPV vaccination (score 1–5)	4.17±0.74	4.26±0.57	−0.43 (0.671)
Not at all (1)	1 (1.4)	0 (0)	
Not likely (2)	0 (0)	0 (0)	
Don’t know (3)	8 (11.4)	3 (6.5)	
Fairly likely (4)	38 (54.3)	28 (60.9)	
Very likely (5)	23 (32.9)	15 (32.6)	
HPV knowledge	*n* (%); correct answer or mean±SD		*χ* ^2^ or *Z* ^*^ (*p*)
High-risk HPV causes STDs (F)	12 (17.1)	15 (32.6)	3.72 (0.156)
Low-risk HPV causes genital cancer (F)	33 (47.8)	19 (42.2)	0.52 (0.770)
Genital warts always cause pain (F)	30 (42.9)	23 (50.0)	0.62 (0.735)
Genital warts cause cervical cancer (F)	20 (28.6)	14 (31.1)	0.20 (0.904)
Cervical cancer is preventable (T)	55 (79.7)	38 (84.4)	0.41 (0.816)
Genital warts are preventable (T)	57 (81.4)	37 (84.1)	1.63 (0.444)
HPV vaccine prevents STD (T)	55 (79.7)	39 (86.7)	0.93 (0.629)
HPV vaccine prevents cervical cancer (T)	53 (75.7)	38 (84.4)	2.04 (0.360)
HPV knowledge score (0–8)	4.58±2.26	4.95±1.89	0.80 (0.373)
Cronbach’s α for eight items=0.85			

^*^Mann–Whitney *U* test.

F, false; T, true.

**Table 5:** **Gender comparisons of correlations among health-belief variables, HPV knowledge, and intention to obtain the HPV vaccination at posttest**

**Variables**	**Intention to obtain HPV vaccination**	
	**Boys**	**Girls**
	**Spearman’s rho (** ***p*** **)**	
Health-belief variables at posttest		
Severity of STDs	0.38 (0.001)	0.49 (0.001)
Severity of cancer	0.30 (0.013)	0.36 (0.015)
Intention to obtain injection for STD prevention	0.85 (<0.001)	0.85 (0.001)
Intention to obtain injection for cancer prevention	0.21 (<0.001)	0.79 (<0.001)
Concern about pain at vaccine injection^a^	0.18 (0.131)	0.40 (0.006)
Benefit of STD prevention injection	0.60 (<0.001)	0.43 (0.003)
Benefit of cancer prevention injection	0.64 (<0.001)	0.53 (<0.001)
HPV knowledge^b^		
High-risk HPV causes STD	0.03 (0.796)	0.07 (0.639)
Low-risk HPV causes genital cancer	0.19 (0.128)	0.00 (0.989)
Genital warts always cause pain	0.21 (0.078)	0.15 (0.312)
Genital warts cause cervical cancer	0.04 (0.774)	0.18 (0.238)
Cervical cancer is preventable	0.12 (0.319)	0.16 (0.282)
Genital warts are preventable	0.23 (0.054)	−0.03 (0.856)
HPV vaccine prevents STDs	0.23 (0.060)	0.18 (0.251)
HPV vaccine prevents cervical cancer	0.25 (0.039)	0.31 (0.037)

^a^Reverse coding; ^b^dummy variables (0, incorrect answer or don’t know; 1, corrected answer).

STD, sexually transmitted disease; HPV, human papillomavirus.

## Discussion

This is the first study to assess the HPV awareness among Korean youth, and to test a preliminary HPV prevention education designed for fifth- and sixth-grade students. This study found that the HPV education not only reduced gender differences with respect to awareness of STDs and cancer, and related health beliefs, but also improved those factors for both genders.

One study conducted in a Western country confirmed that the provision of focused, timely, and ongoing education in the school setting increased HPV vaccination rates and decreased HPV-related morbidity [2]. In addition, the early inclusion of HPV prevention in the health-education curriculum at the elementary-school level has been validated [12].

As mentioned above, the circumstances surrounding HPV vaccination and HPV education in Korean schools are different from those in Western society, with both being very limited so far. A recent study showed that contrary to the observed reduction in the rate of cervical cancer, the rate of HPV-related head and neck cancers is increasing in Korea, as it is in the USA [26]. Furthermore, high-risk HPV infections such as HPV 16, HPV 33, and HPV 18 were very strongly detected in association with male genital warts; this finding was attributed to the changed sexual behaviors of the younger generation in Korea [27]. The adolescent fertility rate, which is one of the established gender-inequality indexes, reportedly increased from 2.3% in 2011 to 5.5% in 2012 [28]. It therefore appears that the instigation of HPV education for both genders of Korean youth would be a timely intervention.

As expected, awareness of HPV was very low among Korean girls and boys, as has been reported in other countries [9,11]. However, the data obtained in this study were limited to a single school setting, and there are no other comparable data on HPV awareness for fifth-grade students. Therefore, future studies should make region and culture comparisons.

It is noteworthy that HPV knowledge acquisition, and specifically the knowledge that “HPV vaccine prevents cervical cancer,” was positively associated with the intention to obtain the HPV vaccination among both girls and boys. In addition, the education session changed the students’ perception of STDs, so that they ultimately saw it as being more severe than they had previously thought, and they became more aware of the benefit of preventing STDs and cancer, so that their intention toward STD prevention was more positive. The necessity for the involvement of men is strongly recommended to improve the perception and intention to prevent HPV [13,14,22,25,29]. The findings of the present study validate the gender-based HPV prevention approach, showing that it is also suitable for elementary students, among whom the gender differences in STDs and cancer awareness disappeared after HPV education.

Gender differences were found at posttest. There was an increase in the intention to submit to an injection for STD prevention among the boys, but not in the intention to submit to an injection for cancer prevention. The education appeared to encourage the boys more toward STD prevention than cancer prevention (i.e., penile, oral, and anal cancers). However, there was an increase in the intention to obtain injections to protect against both STDs and cancer among the girls, illustrating that preadolescent girls were motivated by the education strategy to prevent both STDs and cancer (i.e., cervical cancer). Moreover, the girls were more concerned about the pain at injection than the boys, and their concern about this pain was not reduced by the HPV education. Therefore, future studies should investigate more sensitive approaches for providing information about the subject to girls. Based on the results of the present study, the relationship among rates of HPV infection, HPV vaccination, and cervical cancer should be taught to Korean preadolescent girls and boys. The Korean youth should be supported to recognize and accept not only the concept of HPV vaccination, but also the facts about HPV itself, its potential consequences, and its prevention.

Until now it has been considered that health professionals play a critical role in HPV vaccine recommendation to promote HPV vaccine acceptability, particularly among parents with adolescent children [1,30-34]. One fear among some parents was that the vaccination may give their adolescent children a false sense of security and increase their promiscuity, thereby increasing the probability of an unwanted pregnancy or an STD [35]. Therefore, there is a need for health professionals to provide balanced information about HPV and the HPV vaccination—including the benefits and limitations of the latter—to adolescents and their parents. Strong opinions based on previous studies are that vaccination against HPV should be undertaken in early adolescence, prior to sexual debut, and that an HPV vaccine program should be incorporated into the school vaccination program [36,37]. The role of parents in this issue was not examined in the present study. However, parents could play an indirect role toward educating their children regarding HPV [38,39]. Although they are not yet required to provide permission for their adolescent children to receive the HPV vaccination, since there is as yet no mandatory school vaccine program in Korea, parents could be indirectly informed of the necessity for their children to learn about HPV prevention by providing students with a handout to be taken home. This may increase parental awareness and affect their attitudes toward HPV prevention, including vaccination. If parents support their children in this way, it is expected that both girls and boys would be more knowledgeable and motivated to receive the HPV vaccination in the future when they are eventually faced with the decision regarding whether or not to have it. The role of educating parents regarding HPV and uptake of the HPV vaccination by Korean adolescents should be confirmed in further studies.

This study reconfirmed the effectiveness of the HBM framework to guide HPV prevention [12,14,40]; almost all of the HBM variables were significantly related to intention to obtain the HPV vaccination in both girls and boys. Thus, the HBM framework could be applicable to other areas of sexual health promotion for youth. With respect to the HBM constructs applied in this study, self-efficacy was not examined, despite its prominent role in health behaviors. Thus, the extended HBM variables—including cue to action, motivating factors, and self-efficacy—should be confirmed with respect to HPV prevention among Korean adolescents.

The average age at first sexual intercourse of Korean adolescents appeared to be earlier in 2011 (13.6 years) than in 2009 (14.0 years) [7], indicating that sex education for adolescents in Korea should be actively provided prior to their entry to middle school. It is reported in one survey that less than 40% of Korean adolescents responded that they felt the sex education provided at their school was satisfactory [7]. To a certain extent, the health education provided at elementary schools in Korea is dependent upon the teachers’ autonomy and competency [12]. Pregnancy, childbirth, sexual violence, gender equality, and secondary characteristics are topics that are currently included in the sexual health curriculum for fifth and sixth grades; the topics of contraception and STDs tend to be included at the middle-school level in Korea. However, many Korean middle-school students responded that the best time for beginning sexual education is at elementary school [41]. The HPV lectures and a teaching methodology comprising 2 h of education developed in this study could be used as a model for HPV prevention classes at elementary schools in Korea. HPV education should be tailored to the students’ needs and characteristics. A recent study delivered a coaching program to elementary-school fifth- and sixth-grade students with a view to improving their knowledge and attitudes toward sex [42]. The Korean Association of School Health Teachers suggested drawing up guidelines for HPV education for Korean teachers according to the student level so as to better prepare Korean school health teachers with regard to HPV education.

This study was subject to several limitations with respect to its primitive experimental design, lack of a control group, and the possibility of confounding variables such as the onset of menarche or secondary sexual characteristics, which were not controlled for. Furthermore, the positive perceptual changes in awareness of STDs and cancer prevention, the associated attitudes, and actual vaccine uptake were not used as outcome measures for quantifying the efficacy of the provided HPV education. It should also be noted that the current intentions of Korean students regarding HPV vaccination may also have affected their uptake thereof. Finally, this study did not examine the effectiveness of coeducational classes compared to single-sex classes for HPV education among youth; this needs to be identified in further studies.

Unfortunately, no relevant information regarding the rate of HPV infection in Gangneung city was available, and so the data for Kang Won Do were compared with the national average. The results showed that the age-standardized incidence rate of cervical cancer was 6.6 for Kang Won Do in 2010, which is higher than the national average for the same year 5.9 per 100,000 person [43]. Regardless of the data, the education intervention applied in this study represents an example of gender-based sex education for Korean youth, and appears to be the first exposure to such materials for fifth-grade students. It is expected that the implementation of such educational strategies for students will raise their awareness of gender equality in HPV prevention and increase their motivation and willingness to assimilate the subsequently provided HPV education.

## Conclusions

The findings of this study demonstrate that the implemented 2-h HPV education program was successful for both girls and boys, and could be disseminated to both fifth- and sixth-grade students. Health teachers or school nurses should recognize the concerns and needs that students, parents, and the community have about HPV information, and design an HPV education program that is tailored to these factors.

## Additional file

Additional file 1:
**Human papillomavirus (HPV) infection sheet for primary 5th grade students.** The contents of this handout was modified from “Genital HPV infection-fact sheet” by Centers for Disease Control and Prevention. updated March 20, 2014. Available from http://www.cdc.gov/std/hpv/stdfact-hpv.htm.

## Abbreviations

HPV: Human papillomavirus
STD: Sexually transmitted disease
HBM: Health belief model
