# Supplementary material for: Awareness of human papillomavirus and factors associated with intention to obtain HPV vaccination among Korean youth: quasi experimental study
Source: BMC Int Health Hum Rights. 2015 Feb 21;15:4. doi: 10.1186/s12914-015-0042-2 (PMC4339239; doi:10.1186/s12914-015-0042-2)
Supplement: Additional file 1: — Human papillomavirus (HPV) infection sheet for primary 5th grade students. The contents of this handout was modified from “Genital HPV infection-fact sheet” by Centers for Disease Control and Prevention. updated March 20, 2014. Available from http://www.cdc.gov/std/hpv/stdfact-hpv.htm. [file 12914_2015_42_MOESM1_ESM.docx]

Human papillomavirus (HPV) infection sheet for primary 5^th^ grade students:

1. What is HPV?

HPV is the most common sexually transmitted disease. Some types of HPV can cause genital warts and cancers.

2. What does HPV cause health problems?

By having sex, HPV can be passed even when an infected person has no signs and symptoms. HPV consists of low risk and high risk.

1) Low risk type of HPV: Genital warts appear in the genital area, including anus they can be small or large, raised or flat, or painless or even itching.

2) High risk type of HPV: HPV can cause cervical cancer of women, other cancers, including penis, or anus of men, and oral cancer of both genders.

3. Can we prevent or avoid HPV? Yes, we can do.

1) If possible, it is recommended that the sexual intercourse should be delayed until adulthood.

2) If you become sexually active, by using latex condoms the right way when you have sex, it can lower the chance of getting HPV, but condom may not give full protection against getting HPV. Because HPV can infect that are not covered by the condom.

3) Get vaccinated against HPV. HPV vaccines are safe and effective. They can protect males and females against health problems (including cancers) caused by HPV. 00000

4. What are the advantages and disadvantages of HPV vaccination?

Purpose of vaccination is prevention before we get a particular infection or disease. As you know, there are some examples of vaccination, including hepatitis, encephalitis, or flu. The advantages of the HPV vaccine are prevented, which genital warts caused by low risk HPV and cervical, penile, and oral cancer caused by high risk HPV. However, the disadvantages from the vaccinations are inconvenienced for a three shots over 6 month period, discomforts around the injection site, such as swelling and or itching, and costs for vaccination.

HPV vaccines are recommended to both male and female adolescents (In Korea, recommended age groups are: 9-26 years for girls and 9-15 years for boys).

5. Who can prevent the sexually transmitted disease, cervical cancer, penile cancer and oral cancer? : Men and women both can do that.

The methods are:

1) Postponing the sexual intercourse after adulthood

2) Using condom well when you have sex

3) Screening your health regularly after sexually active

4) Get vaccinated against HPV when you get the chance

In conclusion,

It is more important to prevent sexually transmitted disease than that of treatment.

You can prevent the health problems caused by HPV, including STD, genital warts, and cancers of cervical, penile, and oral since you begin puberty.

Note: The contents of this handout was modified from "Genital HPV infection-fact sheet" by Centers for Disease Control and Prevention.

Updated march 20, 2014. Available from

http://www.cdc.gov/std/hpv/stdfact-hpv.htm.
